# ILC2 require cell-intrinsic ST2 signals to promote type 2 immune responses

**DOI:** 10.3389/fimmu.2023.1130933

**Published:** 2023-03-31

**Authors:** Patrycja M. Topczewska, Zoe A. Rompe, Manuel O. Jakob, Anton Stamm, Pierre S. Leclère, Alexandra Preußer, Claudia U. Duerr, Linda Marie Laura Thole, Katja Kotsch, David Artis, Christoph S. N. Klose

**Affiliations:** ^1^ Charité – Universitätsmedizin Berlin, Corporate Member of Freie Universität Berlin and Humboldt-Universität zu Berlin, Department of Microbiology, Infectious Diseases and Immunology, Hindenburgdamm, Berlin, Germany; ^2^ Charité – Universitätsmedizin Berlin, Corporate Member of Freie Universität Berlin and Humboldt-Universität zu Berlin, Department for General and Visceral Surgery, Hindenburgdamm, Berlin, Germany; ^3^ Jill Roberts Institute for Research in Inflammatory Bowel Disease, Friedman Center for Nutrition and Inflammation, Joan and Sanford I. Weill Department of Medicine, Department of Microbiology and Immunology, Weill Cornell Medicine, Cornell University, New York, NY, United States

**Keywords:** ILC2 - group 2 innate lymphoid cell, IL-33 and ST2, type 2 immune response, mucosal immunity, innate immunity

## Abstract

The initiation of type 2 immune responses at mucosal barriers is regulated by rapidly secreted cytokines called alarmins. The alarmins IL-33, IL-25 and TSLP are mainly secreted by stromal and epithelial cells in tissues and were linked to chronic inflammatory diseases, such as allergic lung inflammation, or to resistance against worm infections. Receptors for alarmins are expressed by a variety of immune cells, including group 2 innate lymphoid cells (ILC2s), an early source of the type 2 cytokines, such as IL-5 and IL-13, which have been linked to atopic diseases and anti-worm immunity as well. However, the precise contribution of the IL-33 receptor signals for ILC2 activation still needs to be completed due to limitations in targeting genes in ILC2. Using the newly established *Nmur1* ^iCre-eGFP^ mouse model, we obtained specific conditional genetic ablation of the IL-33 receptor subunit ST2 in ILC2s. ST2-deficient ILC2s were unresponsive to IL-33 but not to stimulation with the alarmin IL-25. As a result of defective ST2 signals, ILC2s produced limited amounts of IL-5 and IL-13 and failed to support eosinophil homeostasis. Further, ST2-deficient ILC2s were unable to expand and promote the recruitment of eosinophils during allergic lung inflammation provoked by papain administration. During infection with *Nippostrongylus brasiliensis*, ILC2-intrinsic ST2 signals were required to mount an effective type 2 immune response against the parasite leading to higher susceptibility against worm infection in conditional knockout mice. Therefore, this study argues for a non-redundant role of cell-intrinsic ST2 signals triggering proper activation of ILC2 for initiation of type 2 immunity.

## Introduction

Barrier surfaces allow for vital exchange with the environment but are also exposed to a broad array of stressors and are exploited by pathogens for invasion. In case the physical, chemical and biological measures to protect the epithelium from invasion do not prevail, the penetration of the pathogen triggers the secretion of danger signals, such as Interleukin (IL)-33, IL-25 and thymic stromal lymphopoietin (TSLP). Such ‘alarming’ signals activate tissue-resident immune cells and initiate the combat against the pathogen (1). Alarmins are produced to a large degree by specialized epithelial, endothelial or stromal cells in tissue. Recent research has exposed stromal cells as the predominant source of IL-33 in tissues, but dendritic cells, mast cells, endothelial and epithelial cells have been described as IL-33 producers as well (2–6). IL-33 has a chromatin binding motif and nuclear localization sequence, which targets the cytokine in the nucleus. Cell death and cellular stress appear to trigger the release of IL-33 from the nucleus, but the entire process is poorly understood (7). Further processing of full-length IL-33 by various proteases, including allergic proteases, significantly increases its biological activity (8).

Expression of the IL-33 receptor was reported from many immune cells, including innate lymphoid cells type 2 (ILC2s), CD4^+^ and CD8^+^ T cells, basophils, mast cells, eosinophils and macrophages (3, 7, 9–13). The IL-33 receptor is a member of the IL-1 receptor family, which in addition includes IL-1, IL-18 and IL-36 receptors. The binding of IL-33 to the ST2 chain (*Il1rl1*) enables the association with the IL-1rap chain, recruitment of the Myd88 adapter for signal transduction and triggering of a signaling cascade which ultimately leads to the activation of NF-κB as the main transcription factor promoting inflammation (7).

Tissue-resident immune cells include innate lymphoid cells, which are enriched at barrier surfaces and which have a similar functional diversity to T cells in terms of lineage-specifying transcription factors and effector functions. The transcription factors GATA-3, GfI-1, RORα and Bcl11b are crucial for ILC2 development and function in general (14, 15). In addition, ILC2 require the lymphoid cytokine IL-7 to develop (9) and ILC2 activation is regulated by a variety of cytokines, inflammatory mediators, neuronal factors, metabolites and hormones. Upon stimulation, ILC2s secrete IL-5, IL-9, IL-13 and amphiregulin as main effector cytokines to regulate the type 2 immune response in tissues (14, 15).

Single nucleotide polymorphisms (SNPs) in the IL-33 gene have been linked to allergic asthma and chronic inflammatory diseases (16, 17). IL-33 plays an essential role in triggering type 2 immune responses in mouse models of lung inflammation (18, 19). However, since ST2 is expressed by many type 2 effector cells, the precise contribution for ILC2 activation and the initiation of type 2 inflammation remains incompletely understood. This study provides evidence for a cell-intrinsic requirement of ST2 in ILC2s in steady state, as well as models of allergic lung inflammation and anti-worm immunity. By genetically ablating ST2 in ILC2s using *Nmur1* ^iCre-eGFP^ mice, we establish a conditional knockout model to investigate the role of ST2 in ILC2 biology. ST2-deficient ILC2s produced limited amounts of type 2 cytokines and, therefore, did not provide sufficient support for proper eosinophil homeostasis. Furthermore, *Nmur1* ^iCre-eGFP^ *Il1rl1* ^flox/flox^ failed to recruit eosinophils during allergic lung inflammation provoked by papain treatment and mount a fully protective type 2 immune response against *Nippostrongylus brasiliensis* (*N. brasiliensis)* infection. Taken together, our data demonstrate a cell-intrinsic and non-redundant role for ST2 as a regulator of type 2 immunity *via* activation of ILC2s.

## Results

### Genetic ablation of ST2 using *Nmur1* ^iCre-eGFP^ renders ILC2 unresponsive to IL-33

**Figure 1:**
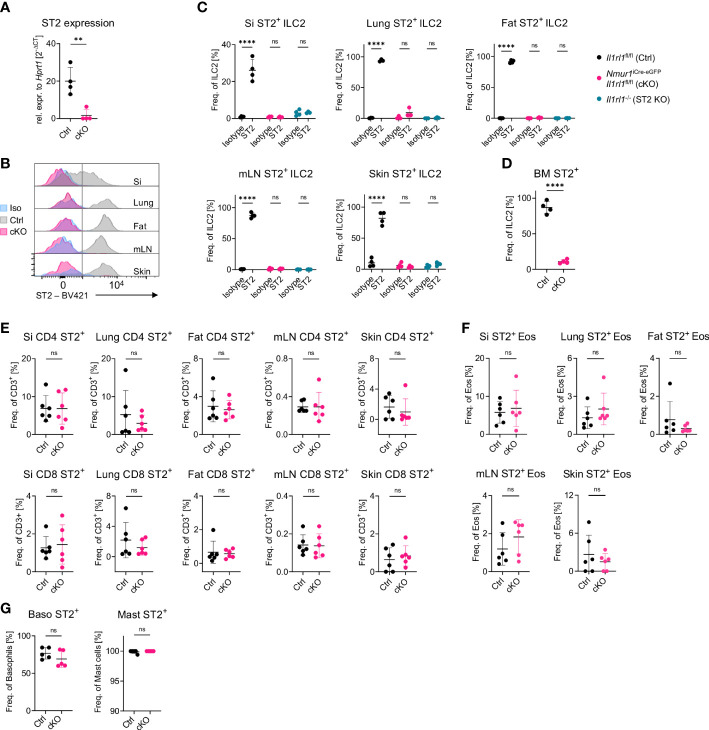
A model for ILC2-specific genetic ablation of ST2. **(A)**, Expression of the *Il1rl1* gene in *Il1rl1* ^flox/flox^ (Ctrl) and *Nmur1* ^iCre-eGFP^ *Il1rl1* ^flox/flox^ (cKO) sort-purified ILC2s. **(B)**, Flow cytometric histograms of ST2 expression in ILC2s of *Il1rl1* ^flox/flox^ (Ctrl) and *Nmur1* ^iCre-eGFP^ *Il1rl1* ^flox/flox^ (cKO) mice across organs, including isotype control on *Il1rl1* ^flox/flox^ (Iso) in the steady state. ILC2s were gated on live CD45^+^ Lin^-^ (CD3, CD5, CD19, Ly6G, Fcϵr1), CD127^+^ and KLRG1^+^. **(C–G)** Percentage of ST2^+^ expression in *Il1rl1* ^flox/flox^ (Ctrl), *Nmur1* ^iCre-eGFP^ *Il1rl1* ^flox/flox^ (cKO) **(D–G)** and *Il1rl1* ^-/-^ (ST2 KO) mice including isotype control **(C)** across different organs in the indicated immune cells. Basophils from spleen and mast cells from the peritoneal lavage. For **(C–G)**: Each symbol represents data from one mouse, data are representative of two experiments with four to six mice per group. Mean +/- SD, Student’s t-Test. ns, not significant, **p < 0.01, ****p < 0.0001.

In order to investigate the importance of the IL-33 receptor for ILC2 regulation *in vivo*, we aimed to target the ST2 chain of the IL-33 receptor (encoded by the *Il1rl1* gene) in ILC2 using the Cre-loxP system. To this end, we crossed the recently developed BAC-transgenic *Nmur1* ^iCre-eGFP^ mouse, which allows gene targeting in ILC2 (20, 21), to *Il1rl1* ^flox/flox^ mice (22). Indeed, *Nmur1* ^iCre-eGFP^ *Il1rl1* ^flox/flox^ lacked ST2 mRNA and protein expression in ILC2s of the small intestine, lung, adipose tissue, mesenteric lymph node, and skin similar to *Il1rl1* ^-/-^ mice but not in CD4^+^ T cells, CD8^+^ T cells, eosinophils, basophils or mast cells (**Figures 1A–G**; **Supplementary Figures 1A–E**).

**Figure 2:**
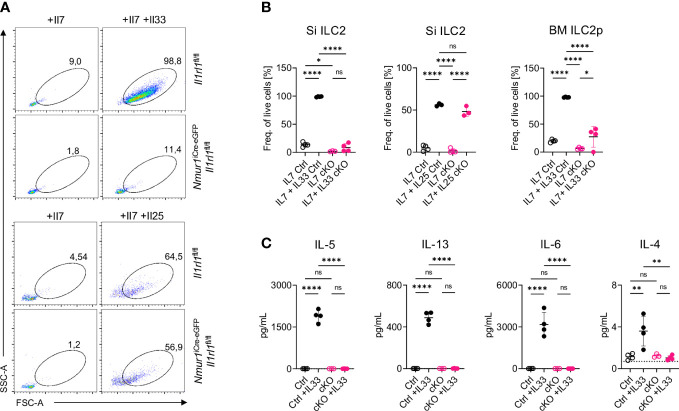
ILC2 of *Nmur1* ^iCre-eGFP^ *Il1rl1* ^flox/flox^ mice are unresponsive to IL-33. **(A)**, Flow cytometric plots of small intestine ILC2s from *Il1rl1* ^flox/flox^ and *Nmur1* ^iCre-eGFP^ *Il1rl1* ^flox/flox^ mice after stimulation with IL-7, IL-7 and IL-33 or IL-7 and IL-25. ILC2s were gated on live CD45^+^ Lin^-^ (CD3, CD5, CD19, Ly6G, Fcer1), CD127^+^ and KLRG1^+^. **(B)**, Quantification of ILC2 activation from **(A)** and bone marrow ILC2s of *Il1rl1* ^flox/flox^ (Ctrl) and *Nmur1* ^iCre-eGFP^ *Il1rl1* ^flox/flox^ (cKO) mice after a three day culture with cytokines IL-7 or IL-7, IL-33 or IL-7, IL-25. **(C)**, Concentration of the indicated cytokine as determined in the cell culture supernatant 3 days after stimulation as in **(A, B)**. For **(B, C)**: Each symbol represents data from one mouse, data are representative of two experiments with four mice per group. Mean +/- SD, One-way ANOVA with multiple comparison. ns, not significant, *p < 0.05, **p < 0.01, ****p < 0.0001.

To validate whether ST2-deficient ILC2s lost the capacity to sense IL-33, we sort-purified ILC2s from the small intestine, which have low ST2 expression, and from the bone marrow, which have high ST2 expression (**Figure 1D**), and cultured the cells with IL-7 alone to provide a survival signal, or IL-7 + IL-33 to induce activation. While control ILC2s massively expanded, blasted and secreted type 2 effector cytokines upon IL-33 stimulation, ST2-deficient ILC2s failed to respond to IL-33, indicating efficient deletion of ST2 and unresponsiveness to react to the ligand (**Figures 2A–C**; **Supplementary Figure 2A**). Next, we aimed to test if ILC2 still properly responded to stimulation with other alarmins despite the conditional deletion of ST2. To this end, we performed a similar *in vitro* assay with sort-purified ILC2s from the small intestine but now exposed the cells to IL-7 or IL-7 + IL-25. ST2-deficient ILC2s expanded and blasted to a comparable degree to ST2-sufficient ILC2s upon IL-25 stimulation (**Figures 2A, B**). We also exposed ILC2s to combinations of TSLP together with IL-7 or IL-25 and IL-33 and assessed blasting and cytokine production of ILC2s (**Supplementary Figures 2B–D**). However, ST2-deficient ILC2s could still respond to TSLP stimulation. Taken together, these data suggest that ILC2 were selectively unresponsive to IL-33 but could still respond to other alarmins, such as IL-25 and TSLP.

### ILC2s develop in comparable proportions without ST2 but have reduced PD-1 expression despite limited phenotypic changes

**Figure 3:**
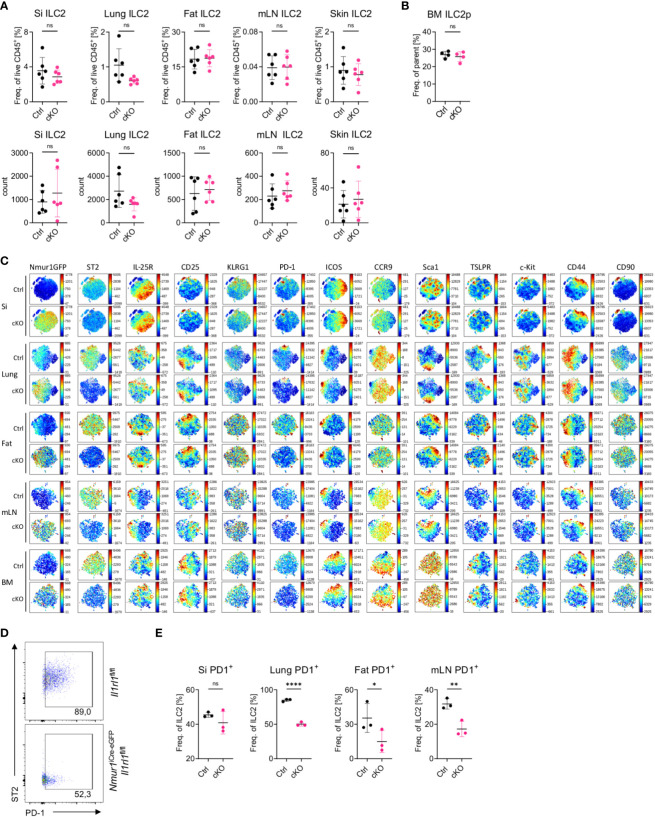
ILC2 develop in normal proportions without ST2 but have reduced PD-1 expression **(A)**, Quantification of relative and absolute ILC2 numbers in *Il1rl1* ^flox/flox^ (Ctrl) and *Nmur1* ^iCre-eGFP^ *Il1rl1* ^flox/flox^ (cKO) mice across different organs in steady state. **(B)**, Quantification of relative ILC2 progenitor numbers in *Il1rl1* ^flox/flox^ (Ctrl) and *Nmur1* ^iCre-eGFP^ *Il1rl1* ^flox/flox^ (cKO) mice in the BM. **(C)**, t-distributed stochastic neighbour embedding plots showing the expression level of different ILC2 markers. ILC2s from naïve *Il1rl1* ^flox/flox^ (Ctrl) and *Nmur1* ^iCre-eGFP^ *Il1rl1* ^flox/flox^ (cKO) mice are shown. Each dot represents a single cell. Data are representative of two experiments with four to five mice per group. **(D)**, Flow cytometric plots of PD-1 expression in lung ILC2s from *Nmur1* ^iCre-eGFP^ *Il1rl1* ^flox/flox^ and *Il1rl1* ^flox/flox^. **(E)**, Quantification of PD-1 expression in *Il1rl1* ^flox/flox^ (Ctrl) and *Nmur1* ^iCre-eGFP^ *Il1rl1* ^flox/flox^ (cKO) mice across different organs in steady state. For **(A, B, D)**: Each symbol represents data from one mouse, data are representative of two experiments with three to eight mice per group. Mean +/- SD, student’s t-Test. ns not significant, *p < 0.05, **p < 0.01, ****p < 0.0001.

Consistent with previous reports investigating mice with germline mutations of ST2, ILC2 were not reduced in frequencies or absolute cell numbers in *Nmur1* ^iCre-eGFP^ *Il1rl1* ^flox/flox^ mice, suggesting that IL-33-ST2 signals are not essential for proper expansion and survival of ILC2s (23, 24) (**Figure 3A**). Similar results were obtained for bone marrow ILC2 progenitors, indicating that lack of ST2 signals did not lead to overt developmental defects (**Figure 3B**) (25).

To test whether deletion of ST2 altered the heterogeneity of ILC2s in tissues, we performed multicolor phenotyping of ILC2s by flow cytometry. Overall, the heterogeneity of ILC2s in tissue was not perturbed and most surface markers used for ILC2 phenotyping, in particular IL-25R or TSLPR were expressed equally in ILC2s from *Il1rl1* ^flox/flox^ and *Nmur1* ^iCre-eGFP^ *Il1rl1* ^flox/flox^ mice (**Figure 3C**). In contrast, the marker PD-1 was consistently downregulated in ST2-deficient ILC2s (**Figures 3C–E**; **Supplementary Figure 3**). Since PD-1 was described as an activation marker on ILC2s (26), these data suggest that ILC2 fail to terminally mature without ST2 signals. In summary, *Nmur1* ^iCre-eGFP^ *Il1rl1* ^flox/flox^ mice are a suitable model to investigate the role of ST2 for ILC2 function *in vivo*.

### Loss of IL-33 signals on ILC2s results in decreased production of effector cytokines and reduced eosinophil counts

**Figure 4:**
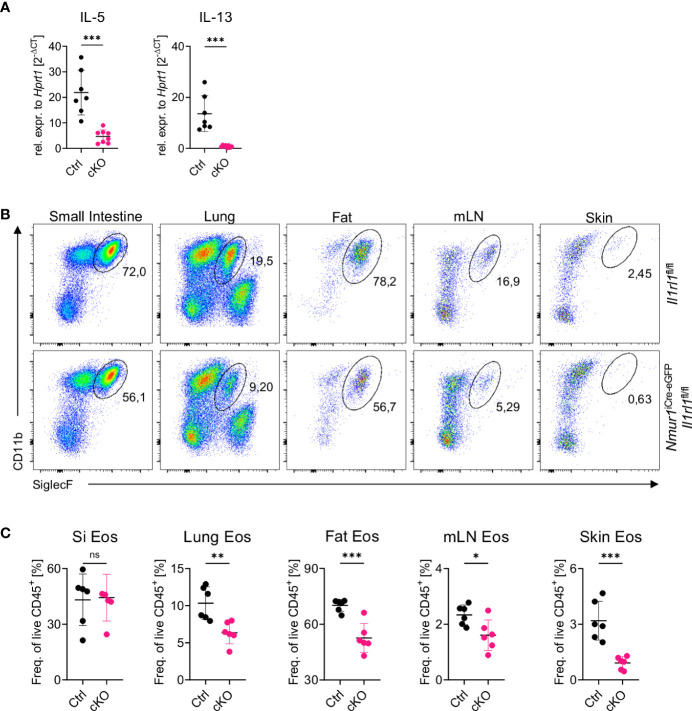
Loss of constant ST2 signaling results in reduced eosinophil counts. **(A)**, Relative expression of the cytokine IL-5 and IL-13 of *Il1rl1* ^flox/flox^ (Ctrl) and *Nmur1* ^iCre-eGFP^ Il1rl1^flox/flox^ (cKO) mice from the lung at steady state. **(B)**, Flow cytometric plots of eosinophils from *Il1rl1* ^flox/flox^ and *Nmur1* ^iCre-eGFP^ *Il1rl1* ^flox/flox^ mice across different organs in steady state. Eosinophils were gated on live CD45^+^ Lin^-^ (CD3, CD5, CD19), CD11b^+^ and SiglecF^+^. **(C)**, Quantification of **(B)** in *Il1rl1* ^flox/flox^ (Ctrl) and *Nmur1* ^iCre-eGFP^ *Il1rl1* ^flox/flox^ (cKO) mice across different organs in steady state. For **(A, C)**: Each symbol represents data from one mouse, data are representative of two experiments with five to seven mice per group. Mean +/- SD, student’s t-Test. ns, not significant, *p < 0.05, **p < 0.01, ***p < 0.001.

Several publications have highlighted the essential role of ILC2 and ILC2-derived IL-5 for eosinophil homeostasis (21, 27). Additional evidence suggests that IL-5 secretion is under the control of alarmin signals (23, 28) and that PD-1^+^ ILC2 are potent cytokine producers (26). Therefore, we asked if ST2 signals are required for type 2 cytokine production by ILC2. Indeed, IL-5 and IL-13 mRNA levels were significantly reduced in sort-purified ILC2s from *Nmur1* ^iCre-eGFP^ *Il1rl1* ^flox/flox^ compared to control ILC2s (**Figure 4A**). Next, we assessed the number of eosinophils in *Nmur1* ^iCre-eGFP^ *Il1rl1* ^flox/flox^ mice at steady state due to the IL-5 cytokine being crucial for eosinophil homeostasis (29, 30). Consistent with reduced IL-5 levels in ILC2s, we detected a significant reduction of eosinophils in the lung, adipose tissue, mesenteric lymph nodes and skin, suggesting that ILC2-intrinsic sensing of IL-33 *via* ST2 is necessary for type 2 cytokine production regulating proper eosinophil homeostasis (**Figures 4B, C**).

### ILC2-intrinsic ST2 controls eosinophil recruitment during allergic lung inflammation

**Figure 5:**
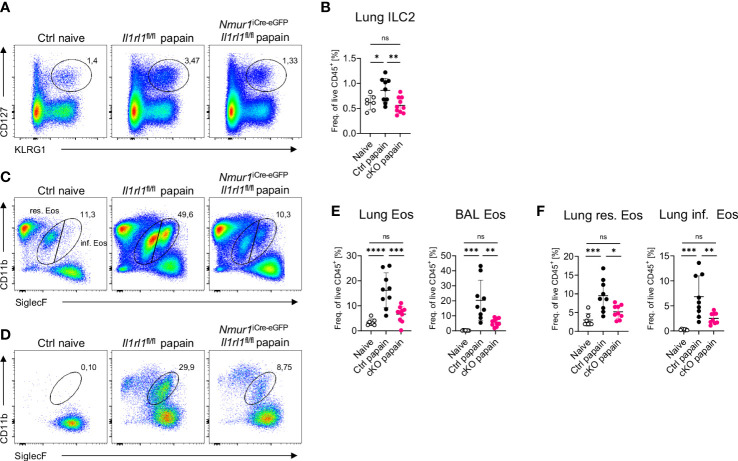
ST2-deficient ILC2s fail to recruit eosinophils in acute lung inflammation. **(A, C, D),** Flow-cytometric plots showing ILC2s **(A)** in the Lung, eosinophils **(C)** in the lung or BAL **(D)** of untreated Ctrl (naïve) animals and papain treated *Nmur1* ^iCre-eGFP^ *Il1rl1* ^flox/flox^ mice (cKO) and littermate controls (Ctrl). Data are representative of two experiments with four to six mice per group. **(B, E)**, Quantification of relative ILC2 **(B)** and eosinophil numbers from the lung or BAL **(E)** in the lung and BAL. **(F)**, Quantification of relative resident and inflammatory eosinophil numbers in the lung. For **(B, E, F)**: One-way ANOVA with multiple comparisons, ns, not significant, *p < 0.05, **p < 0.01, ***p < 0.001, ****p < 0.0001.

Next, we tested how the lack of ST2 on ILC2s influences the type 2 immune responses in the context of allergic lung inflammation, which was provoked by the administration of the protease allergen papain. Papain administration resulted in expansion of ILC2s, which was significantly reduced in *Nmur1* ^iCre-eGFP^ *Il1rl1* ^flox/flox^ mice (**Figures 5A, B**). We also found a strong increase in eosinophil counts in lung and BAL fluid upon papain treatment (**Figures 5C–E**). This increase in eosinophil counts was drastically reduced in papain-treated *Nmur1* ^iCre-eGFP^ *Il1rl1* ^flox/flox^ mice (**Figures 5C–E**). A more detailed analysis revealed that both CD11b^+^ SiglecF^int^ tissue-resident and CD11b^+^ SiglecF^high^ inflammatory eosinophils (31) were reduced in *Nmur1* ^iCre-eGFP^ *Il1rl1* ^flox/flox^ mice, suggesting that ST2 signals in ILC2s are required for recruitment and local proliferation of eosinophils (**Figures 5C, F**).

### *Nmur1* ^iCre-eGFP^ *Il1rl1* ^flox/flox^ mice have a defective type 2 immunity to *N. brasiliensis* infections

**Figure 6:**
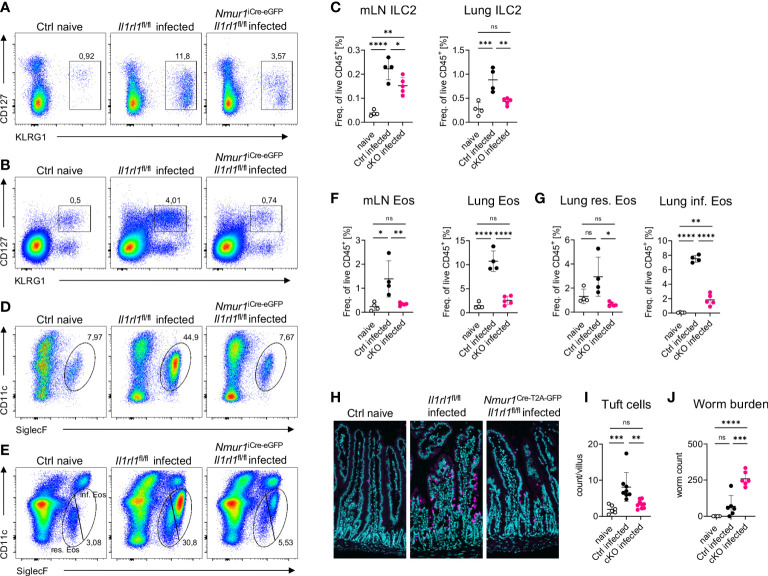
ILC2 intrinsic ST2 is required for immunity against *N. brasiliensis* infection. **(A, B)**, Flow-cytometric plots showing ILC2s in the mLN **(A)** and in the lung **(B)** of untreated Ctrl (naïve) animals and infected *Nmur1* ^iCre-eGFP^ *Il1rl1* ^flox/flox^ mice (cKO) and littermate controls (Ctrl). Mice were infected with *N. brasiliensis*. **(C)**, Quantification of **(A, B)**. **(D, E)** Flow-cytometric plots showing eosinophils in the mLN **(D)** and in the lung **(E)** of untreated Ctrl (naïve) animals and infected *Nmur1* ^iCre-eGFP^ *Il1rl1* ^flox/flox^ mice (cKO) and littermate controls (Ctrl). Mice were infected with *N. brasiliensis*. **(F)**, Quantification of **(D)** and **(E, G)**, Quantification of relative resident and inflammatory eosinophil numbers in the lung of untreated Ctrl (naïve) animals and infected *Nmur1* ^iCre-eGFP^ *Il1rl1* ^flox/flox^ mice (cKO) and littermate controls (Ctrl). **(H)**, Immunofluorescence migrographs of histological sections of the murine small intestine, tuft cells stained with DCLK1 (magenta), DAPI (blue). **(I)**, Quantification of **(H)**. Tuft cell numbers per villus. Data combined from two independent experiments. **(J)**, Worm burden in untreated animals (naïve) and infected *Nmur1* ^iCre-eGFP^ *Il1rl1* ^flox/flox^ mice (cKO) or littermate controls (Ctrl). For **(C, F, G, I, J)**: Data are representative of two experiments with four to six mice per group. One-way ANOVA with multiple comparisons, ns, not significant, *p < 0.05, **p < 0.01, ***p < 0.001, ****p < 0.0001.

To test whether ST2 is required in a cell-intrinsic manner on ILC2s to mediate resistance against helminth infections, we infected *Nmur1* ^iCre-eGFP^ *Il1rl1* ^flox/flox^ and littermate control mice with the worm *N. brasiliensis*. Littermate control mice mounted a potent type 2 immune response upon worm infection, which was diminished in *Nmur1* ^iCre-eGFP^ *Il1rl1* ^flox/flox^ mice (**Figure 6**). The massive expansion of ILC2s upon infection was weakened in *Nmur1* ^iCre-eGFP^ *Il1rl1* ^flox/flox^ mice compared to littermate controls (**Figures 6A–C**). Furthermore, eosinophil frequencies and tuft cell hyperplasia were reduced in conditional knockout mice, underlining the importance of ST2 signals for a proper type 2 immune response (**Figures 6D–I**). As a consequence, *Nmur1* ^iCre-eGFP^ *Il1rl1* ^flox/flox^ mice had higher worm counts in the intestine (**Figure 6J**). Therefore, our data expose cell-intrinsic ST2 signals in ILC2s as a non-redundant stimulatory pathway for triggering protective type 2 immunity.

## Discussion

Alarmins are important mediators of inflammation and molecules targeting these pathways have already been or are about to be tested in clinical trials (32–35). However, since alarmins are sensed by a variety of immune cells, the mechanism of how they modulate immune response is incompletely understood. In this study, we characterized the IL-33-ST2 signaling pathway for ILC2 activation in detail, which could not be addressed so far because of limitations in genetic targeting strategies, using a novel Cre deleter strain based on the *Nmur1* promoter.

IL-33 and the type 2 effector cytokines IL-4, IL-5 and IL-13 have been linked to atopic diseases in mice and humans (16, 17). Similarly, type 2 effector cells, such as ILC2, Th2, mast cells and basophils were linked to this disease entity and were reported to respond to IL-33 with the release of type 2 effector cytokines and are therefore a potential candidate to mediate the effects of IL-33 in the context of type 2 inflammation. In our study interrogating the role of ST2 on ILC2s, we found that conditional deletion of ST2 rendered ILC2s unresponsive to IL-33 and resulted in decreased production of the effector cytokines IL-5 and IL-13 directly *ex vivo*. In contrast, ILC2s developed in regular proportions without ST2. These findings are consistent with several studies, which found that the germline deletion of the alarmin receptors IL-25, IL-33 and TSLP did not result in decreased ILC2 numbers but diminished cytokine expression (23, 24). We further detected a reduction in eosinophil numbers at steady state in *Nmur1* ^iCre-eGFP^ *Il1rl1* ^flox/flox^ mice, which is most likely the consequence of diminished IL-5 production by ILC2s, since ILC2s were described being the major source of IL-5 in tissues (27) and the IL-5–IL-5ra signaling was proven indispensable for proper development of eosinophils (29, 30). Finally, ILC2-deficient mice have defects in eosinophil development comparable to the deficiency described in IL-5 or IL5rα knockout mice (21, 29, 30). Of note, similar effects on eosinophil numbers were found in mice with germline deletion of ST2 combined with recombinant IL-33 administration (36). The results of our study allow to conclude that ILC2s are the cell type requiring IL-33 signals *via* ST2 in order to regulate eosinophil homeostasis at steady state and during allergic lung inflammation.

Despite the well-established role of alarmins in triggering allergic lung inflammation, the target cell types are only partly defined. IL-33 receptor was found to play a pivotal role in activating different effector cells in the context of type 2 inflammation including T_H_2 cells, eosinophils, basophils and mast cells. The contribution of our study is the demonstration of an indispensable role for the IL-33-ST2 axis to stimulate ILC2 activation during allergic lung inflammation, which cannot be compensated by other signaling pathways or cell types. While we did not examine the role of conditional deletion of ST2 in other type 2 immune cells, our data suggest that IL-33 primarily activates ILC2 to initiate type 2 inflammation.

We used the *Nmur1* ^iCre-eGFP^ *Il1rl1* ^flox/flox^ mouse model to obtain genetic deletion of ST2 in ILC2s but not in other cell types investigated (**Figures 1A–G**). The *Nmur1* ^iCre-eGFP^ mice have reported highly efficient recombination capabilities (20, 21). To confirm the efficient deletion of ST2, we have compared ST2 levels to isotype control and mice with germline mutation of ST2. While very efficient deletion was obtained in most organs as validated by flow cytometry, a minor fraction of cells remained formally ST2^+^ in the lung of *Nmur1* ^iCre-eGFP^ *Il1rl1* ^flox/flox^ mice, which might be explained by the limitations in the discriminatory power of the ST2 antibody staining (**Figures 1B, C**).

Our results with respect to ST2 signaling in ILC2s are comparable to published studies investigating the effect of conditional gene deletion of alarmin receptors in ILC2s. Kabata et al. could show that TSLP receptor is required on ILC2s to trigger allergic lung inflammation provoked by papain using *Il7r* ^Cre/+^ and *Il5* ^Cre/+^ and conditional alleles of TSLP receptor. The same study described a pivotal role of TSLP receptor on dendritic cells and CD4^+^ T cells in the ovalbumin-induced airway inflammation model (37). Furthermore, Leyva-Castillo et al. conditionally deleted the IL-25 receptor (IL-25R) by crossing *Rora* ^Cre/+^ and *IL17rb* ^flox/flox^ mice. In a model of skin inflammation provoked by ova treatment, the symptoms were reduced in *Rora* ^Cre/+^ and *IL17rb* ^flox/flox^ mice, suggesting a crucial role for the IL-25R for ILC2 activation (38).

Previous studies reported a pivotal role for IL-25 and IL-33 in regulating worm expulsion of the parasites *N. brasiliensis* and *Strongyloides venezuelensis* (9, 28, 39–41). IL-25 together with other inflammatory mediators is secreted by Tuft cells and activates a subsets of IL-25R^+^ ST2^-^ ILC2s coined inflammatory ILC2s (42–46). IL-33 was also reported to stimulate the generation of inflammatory ILC2s *via* activation of the enzyme tryptophan hydroxylase in ILC2s (43, 47). A correlation of ILC2 frequencies and worm resistance during *N. brasiliensis* infection further supports the idea that ILC2s have a crucial function for worm expulsion, most likely activated by alarmin signals. Adoptive transfer experiments of ILC2s in lymphopenic or immunodeficient mice did rescue defects to expel the parasite, and therefore provide additional evidence for a pivotal role of ILC2s (9, 28). Complete depletion of ILC2 using *Nmur1* ^iCre-eGFP^ *Ild2* ^flox/flox^, *Nmur1* ^iCre-eGFP^ *Gata3* ^flox/flox^ or *Klrg1* ^Cre^ *Gata3* ^flox/flox^ resulted in a severe defect in worm expulsion demonstrating the essential role of ILC2 for resistance to *N. brasiliensis* infection (21, 48). IL-25R and ST2 fulfill complementary functions to trigger ILC2 activation since genetic ablation of both receptors resulted in a comparable phenotype to ILC2-deficient mice characterized by strongly delayed worm expulsion and ongoing worm infection until day 14 - 20 post infection (28).

The *Nmur1* ^iCre-eGFP^ *Il1rl1* ^flox/flox^ mouse model allows us to more directly address the question of ILC2 redundancy and IL-33 signals without using adoptive cell transfer systems in combination with lymphopenic mice. Overall, our data from the *Nmur1* ^iCre-eGFP^ *Il1rl1* ^flox/flox^ mice establish a cell-intrinsic and non-redundant role of ST2 for ILC2 function in the context of a type 2 immune response in lymphoreplete mice.

## Experimental procedures

### Mouse strains

C57BL/6 mice were purchased from Janvier. *Il1rl1* ^flox/flox^ mice (22) (provided by the UC Davis/MMRRC repository), *Il1rl1* ^-/-^ mice (28) and *Nmur1* ^iCre-eGFP^ mice (20, 21) bred locally at Charité. Sex and age-matched animals were used for experiments if not otherwise indicated. We did not use randomization to assign animals to experimental groups. All animal experiments were approved and are in accordance with the local animal care committees (Lageso Berlin).

### Cell isolation

Small intestine was removed, cleaned from remaining fat tissue and washed in ice-cold PBS. Peyer’s patches were eliminated, small intestine was opened longitudinally and washed in ice-cold PBS. Dissociation of epithelial cells was performed by incubation on a shaker at 37°C in HBSS (Sigma-Aldrich) containing 10 mM Hepes (Gibco) and 5 mM EDTA (Roboklon) two times for 15 min. After each step, samples were vortexed and the epithelial fraction discarded. Afterwards, remaining tissue was chopped into small pieces and enzymatic digestion was performed using dispase (0,5 U/ml; Corning), collagenase D (0,5 mg/ml; Roche) and DNaseI (100 μg/ml; Sigma-Aldrich). Leukocytes were further enriched by Percoll gradient centrifugation (GE Healthcare). Lungs were chopped and incubated in the enzyme cocktail described above for 40 min on a shaker at 37°C. The remaining tissues were mashed with a syringe plunger and single cell suspensions were filtered through a 70 μm cell strainer. Leukocytes were then further enriched by Percoll gradient centrifugation. Mesenteric lymph nodes were chopped and incubated in RPMI 1640 medium (Gibco) supplemented with 1% BSA (Sigma-Aldrich), collagenase II (1 mg/ml; Sigma-Aldrich) and DNaseI (100 μg/ml) for 20 min on a shaker at 37°C. Afterwards, cells were dissociated using a pasteur pipette, and filtered through a 70 μm cell strainer. Epididymal white adipose tissue was removed and incubated in the same digestion buffer for 45 min on a shaker at 37°C. After incubation, cells were dissociated using a Pasteur pipette, filtered through a 70 μm cell strainer, spun down and the adipocyte layer was aspirated. For skin preparation, ears were removed, split and put dermal side down in DMEM supplemented with Liberase TL (0,5 mg/ml; Roche) at 37°C for 1,5 h. The tissue was then mashed through a 70 μm cell strainer and the leukocytes further enriched by Percoll gradient centrifugation. For isolation of bone marrow cells, femur and tibia bone were crushed with a pestle, rinsed and cells were filtered through a 70 μm cell strainer. Red cell lysis was performed in ACK lysis buffer for 3 min.

### Flow cytometry and cell sorting

Dead cells were routinely excluded with Fixable Aqua Dead Cell Stain or SYTOX Blue Dead Cell Stain (Thermo Fisher Scientific). Single cell suspensions were incubated on ice with anti-CD16/CD32 antibody and the following conjugated antibodies in PBS (Ca2+ and Mg^2+^-free, Sigma-Aldrich). If indicated, lineage-positive cells were excluded by staining for CD3e (145-2C11 or 500A2), CD5 (53-7.3), CD19 (1D3 or 6D5), FcϵRIα (Mar-1) and Ly6G (1A8). For surface staining the following antibodies were used: c-Kit (2B8), CCR9 (9B1), CD11b (M1/70), CD11c (N418), CD127 (A7R34), CD25 (PCG1.5), CD4 (GK1.5 and RM4-5), CD44 (IM7), CD45.2 (104), CD49b (DX5), CD64 (X54-5/7.1), CD8a (53-6.7), CD90.2 (53-2.1), F4/80 (BM8), ICOS(C398.4A), IL-25R (Munc33), KLRG1 (2F1 or MAFA), Ly6G (1A8), NK1.1 (PK136), PD-1 (29F.1A12), Sca-1 (D7), SiglecF (E50-2440), ST2 (RMST2-33), TSLPR (22H9), Rat IgG2b kappa Isotype (eB149/10H5). All antibodies used in flow cytometry were purchased from eBioscience, Biolegend or BD Biosciences if not otherwise indicated. All flow cytometry experiments were acquired using a custom configuration Fortessa flow cytometer and the FACS Diva software (BD Biosciences) and were analyzed with FlowJo V9.9.3 or V10.6.2 software (TreeStar) and for the t-SNE plots analyzed with the Cytobank Software using custom configurations (Backman Coulter) or sort-purified by using a custom configuration FACSAria cell sorter (BD Biosciences).

### Quantitative real-time PCR

Sort-purified cells were homogenized in Trizol (Thermo Fisher Scientific) and stored at -80°C. RNA was extracted with chloroform and RNA concentration was determined using a Nanodrop 2000 spectrophotometer (Thermo Fisher Scientific). Reverse transcription of total RNA was performed using the High Capacity cDNA Reverse Transcription kit according to the protocol provided by the manufacturer (Thermo Fisher Scientific). Reaction was detected on a QuantStudio 5 Real-Time PCR (Thermo Fisher Scientific) using Taqman Gene Expression Assay (Applied Biosystems) with *Il5* (Mm00439646_m1), *Il13* (Mm00434204_m1) or SYBR Green Master Mix with *Il1rl1* (forward: 5’- GGGCACACAGGTCCTACTTG-3’, reverse: 5’- ATGTAGTTGGTTCCATTCTCCG-3’). Gene expression was normalized to the housekeeping gene *Hprt1* (Mm00446968_m1) for Taqman and *Hprt1* (forward: 5’-GATACAGGCCAGACTTTGTTGG-3’, reverse: 5’-CAACAGGACTCCTCGTATTTGC-3’) for SYBR Green.

### Helminth infection and allergic asthma induction

Third-stage larvae (L3) of *N. brasiliensis* were purified with a Baermann apparatus. After washing three times in PBS, larvae were counted and 500 purified larvae were injected subcutaneously in PBS. Mice were killed, organs were analyzed and worm burden was determined in the small intestine 7 days post infection.

For allergic asthma induction, 30 μg of Papain (Roche) in PBS were administered intranasally on three consecutive days. Mice were killed on day seven after initial administration, organs were collected and analyzed.

### Cell culture

Sort-purified ILC2s (as live CD45^+^, Lin^-^, NK1.1^-^, CD127^+^ and KLRG1^+^ for small intestine or CD25^+^ for bone marrow) were incubated in DMEM with high glucose supplemented with 10% FCS, 10 mM Hepes, 1 mM sodium pyruvate, non-essential amino acids, 80 μM 2-Mercaptoethanol, 2mM Glutamine, 100 U/ml Penicillin and 100 μg/ml Streptomycin (all from Gibco) in 96-well U-bottom microtiter plates (Nunc) for three days at 37°C and 5% CO_2_. As indicated, the culture was supplemented with a cocktail of IL-7 only, IL-7 and IL-33 or IL-7 and IL-25, IL-7 and TSLP or IL-7 and IL-33, IL-25, TSLP (Biolegend, TSLP from R&D, 20 ng/ml each).

### Cytokine measurement

Cytokine concentrations in culture supernatants were determined by using the LEGENDplex murine T_H_2 Panel V03 multiplex beads-based assay (Biolegend) according to the manufacture’s protocol. Samples were recorded on a custom configuration Fortessa flow cytometer and the FACS Diva software (BD Biosciences) and the flow cytometry data files were analyzed using the Legendplex cloud-based analysis software suite (Biolegend).

### Histology and immunofluorescence microscopy

For immunofluorescence staining, the tissue was fixed in 4% PFA at 4°C until tissue embedding. Paraffin-embedded sections were de-paraffinized and rehydrated. Sections were permeabilized with 0.5% Triton-X in PBS and blocked with PBS 0,5% Triton X-100 and 10% serum and stained with rabbit anti-DCLK1 (Abcam) followed by donkey anti-rabbit antibody coupled to Alexa Fluor 555 (Thermo Fisher Scientific). Nuclei were counterstained with DAPI (Thermo Fisher Scientific). Images were captured on a Zeiss Axio Observer 7 microscope and analysed with Zen software (Zeiss). For tuft cell numbers, five to ten representative villi were counted on four independent images per mouse using ImageJ.

### Statistical analysis

Data is plotted showing the mean +/- standard deviation. P values of data sets were determined by unpaired two-tailed Student’s t-test with 95% confidence interval. If equal variances could not be assumed, Welch test was performed. Ordinary one-way ANOVA with Tukey’s multiple comparisons test was used to analyze several groups. Normal distribution was assumed. Before mentioned statistical tests were performed with Graph Pad Prism V9 software (GraphPad Software, Inc.). (*p <0.05; **p <0.01; ***p <0.001; ****p <0.0001 and ns, not significant).

## Data availability statement

The original contributions presented in the study are included in the article/**Supplementary Material** further inquiries can be directed to christoph.klose@charite.de.

## Ethics statement

The animal study was reviewed and approved by Lageso Berlin.

## Author contributions

PMT and ZAR carried out the experiments and analyzed the data. MOJ, PL, AS, AP and CSNK helped performing experiments. LMLT and KK helped with the t-SNE analysis. CUD and DA provided crucial input and tools for the study. CSNK and PMT conceived the project and wrote the manuscript with input from all co-authors. All authors contributed to the article and approved the submitted version.

## Supplementary material

Supplementary Figure 1 (related to **Figure 1**): Gating strategy for flow cytometry. **(A, B)**, Gating strategy of ILC2s and eosinophils from the small intestine, lung, mesenteric lymph nodes, fat, skin **(A)** and bone marrow **(B)**. Lineage: CD3, CD5, CD19, Ly6G, FcϵRIα. **(C)**, Gating strategy of CD4+ and CD8+ T cells from the lung. **(D)**, basophils from the spleen and mast cells from the peritoneal lavage. Lineage: CD3, CD5, CD19, Ly6G. **(E)**, Geometric mean fluorescent intensity measurement of ST2 in ILC2s from *Il1rl1* ^flox/flox^ (Ctrl), *Nmur1* ^iCre-eGFP^ *Il1rl1* ^flox/flox^ (cKO) and *Il1rl1* ^-/-^ (ST2 KO) mice (indicated as ST2) with isotype control (indicated as Isotype) across different organs. For **(A-D)**: *Il1rl1* ^flox/flox^ (Ctrl) and *Nmur1* ^iCre-eGFP^ *Il1rl1* ^flox/flox^ mice (cKO). Mean ± SD. Student's t-Test. ns, not significant, **p < 0.01, ****p < 0.0001.

Supplementary Figure 2 (related to **Figure 2**): ST2-deficient ILC2s are unresponsive to IL-33 but not to other alarmins. (**A)**, Cytokine measurement from culture supernatants three days after stimulation as in (**Figure 2C**) (extending). **(B)**, Flow cytometric plots of ILC2s from the small intestine of *Il1rl1* ^flox/flox^ and *Nmur1* ^iCre-eGFP^ *Il1rl1* ^flox/flox^ mice after stimulation with IL-7, IL-7 and IL-33, IL-7 and Il-25, IL-7 and TSLP or IL-7 and IL-33, IL-25 and TSLP (alrmins) as indicated. ILC2s were gated on live CD45^+^ Lin^-^ (CD3, CD5, CD19, Ly6g, Fcer1), CD127^+^ and KLRG1^+^. **(C)**, Quantification of ILC2 activation from **(B)** of *Il1rl1* ^flox/flox^ (Ctrl) and *Nmur1* ^iCre-eGFP^ *Il1rl1* ^flox/flox^ (cKO) mice after a three day culture with cytokines IL-7, IL-7 and IL-33, IL-7 and Il-25, IL-7 and TSLP or IL-7 and IL-33, IL-25 and TSLP (alrmins) as indicated. **(D)**, Concentration of the indicated cytokine as determined in the cell culture supernatant three days after stimulation as in **(B, C)**. For **(A, C, D)**: Each symbol represents data from one mouse, data are representative of two experiments with four mice per group. Mean +/- SD. One-way ANOVA with multiple comparisons **(A)**, Student’s t-Test **(C, D)**. ns not significant, * p<0.05, ** p<0.01, **** p<0.0001.

Supplementary Figure 3 (related to **Figure 3**): ST2 dependent changes in PD-1 expression. Geometric mean fluorescent intensity measurement of PD-1 in ILC2s from *Il1rl1* ^flox/flox^ (Ctrl) and *Nmur1* ^iCre-eGFP^ *Il1rl1* ^flox/flox^ (cKO) mice across different organs in steady state. Each symbol represents data from one mouse, data are representative of two experiments with three to four mice per group. Mean +/- SD, student’s t-Test. ns not significant, * p<0.05, **** p<0.0001.
