# Supplementary figures and images for: ILC2 require cell-intrinsic ST2 signals to promote type 2 immune responses
Source: Front Immunol. 2023 Mar 31;14:1130933. doi: 10.3389/fimmu.2023.1130933 (PMC10104602; doi:10.3389/fimmu.2023.1130933)

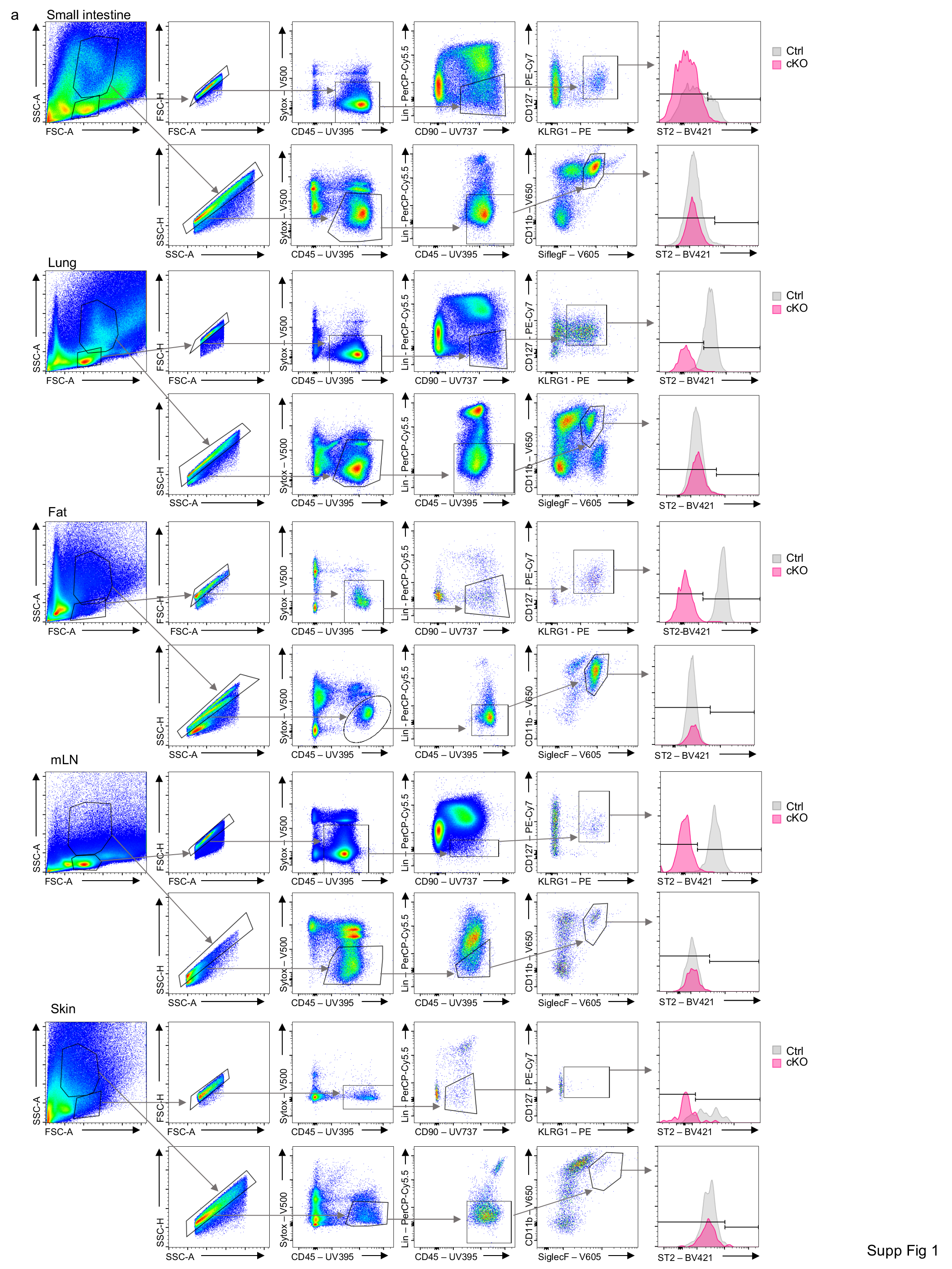

Supplement: Supplementary Figure 1 — (related to Figure 1 ): Gating strategy for flow cytometry. (A, B), Gating strategy of ILC2s and eosinophils from the small intestine, lung, mesenteric lymph nodes, fat, skin (A) and bone marrow (B). Lineage: CD3, CD5, CD19, Ly6G, FcϵRIα. (C), Gating strategy of CD4+ and CD8+ T cells from the lung. (D), basophils from the spleen and mast cells from the peritoneal lavage. Lineage: CD3, CD5, CD19, Ly6G. (E), Geometric mean fluorescent intensity measurement of ST2 in ILC2s from Il1rl1 flox/flox (Ctrl), Nmur1 iCre-eGFP Il1rl1 flox/flox (cKO) and Il1rl1 -/- (ST2 KO) mice (indicated as ST2) with isotype control (indicated as Isotype) across different organs. For (A-D): Il1rl1 flox/flox (Ctrl) and Nmur1 iCre-eGFP Il1rl1 flox/flox mice (cKO). Mean ± SD. Student's t-Test. ns, not significant, **p < 0.01, ****p < 0.0001. [file Image_1.tiff]

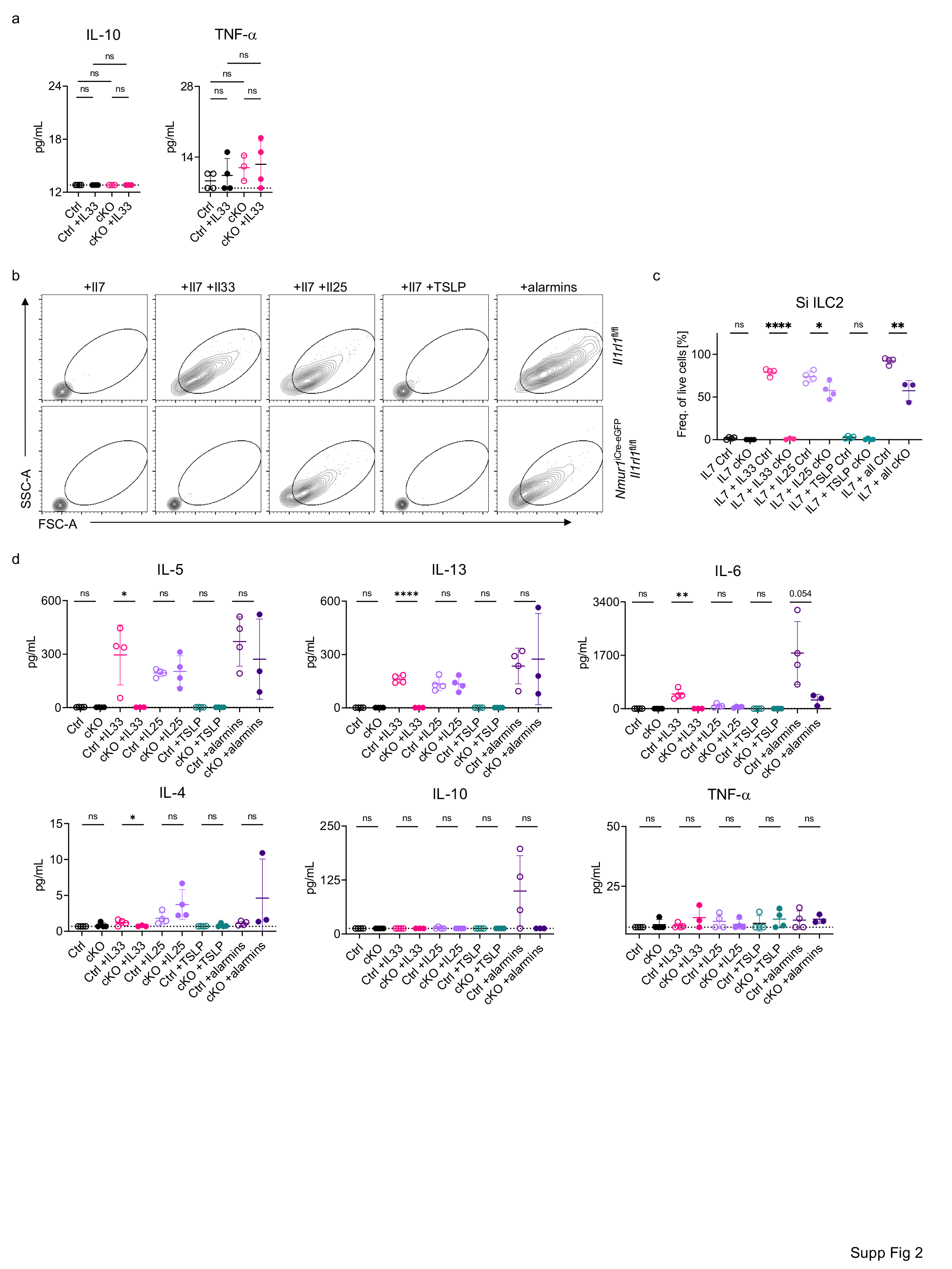

Supplement: Supplementary Figure 2 — (related to Figure 2 ): ST2-deficient ILC2s are unresponsive to IL-33 but not to other alarmins. (A), Cytokine measurement from culture supernatants three days after stimulation as in ( Figure 2C ) (extending). (B), Flow cytometric plots of ILC2s from the small intestine of Il1rl1 flox/flox and Nmur1 iCre-eGFP Il1rl1 flox/flox mice after stimulation with IL-7, IL-7 and IL-33, IL-7 and Il-25, IL-7 and TSLP or IL-7 and IL-33, IL-25 and TSLP (alrmins) as indicated. ILC2s were gated on live CD45+ Lin- (CD3, CD5, CD19, Ly6g, Fcer1), CD127+ and KLRG1+. (C), Quantification of ILC2 activation from (B) of Il1rl1 flox/flox (Ctrl) and Nmur1 iCre-eGFP Il1rl1 flox/flox (cKO) mice after a three day culture with cytokines IL-7, IL-7 and IL-33, IL-7 and Il-25, IL-7 and TSLP or IL-7 and IL-33, IL-25 and TSLP (alrmins) as indicated. (D), Concentration of the indicated cytokine as determined in the cell culture supernatant three days after stimulation as in (B, C). For (A, C, D): Each symbol represents data from one mouse, data are representative of two experiments with four mice per group. Mean +/- SD. One-way ANOVA with multiple comparisons (A), Student’s t-Test (C, D). ns not significant, * p<0.05, ** p<0.01, **** p<0.0001. [file Image_2.tiff]
